# COVID‐19 mRNA vaccine‐related interstitial lung disease: Two case reports and literature review

**DOI:** 10.1002/rcr2.938

**Published:** 2022-03-23

**Authors:** Clara So, Shinyu Izumi, Akane Ishida, Ryo Hirakawa, Yusaku Kusaba, Masao Hashimoto, Satoru Ishii, Hideki Miyazaki, Motoyasu Iikura, Masayuki Hojo

**Affiliations:** ^1^ Department of Respiratory Medicine National Center for Global Health and Medicine Shinjuku City Japan; ^2^ Division of Pathology National Center for Global Health and Medicine Shinjuku City Japan

**Keywords:** COVID‐19, interstitial lung disease, mRNA vaccine, steroid, vaccine‐induced pneumonitis

## Abstract

The Pfizer‐BioNTech mRNA vaccine (BNT162b2) is an effective and well‐tolerated coronavirus disease 2019 (COVID‐19) vaccine. However, rare adverse events have been reported. We report two cases of COVID‐19 mRNA vaccine‐related interstitial lung disease (ILD). A 67‐year‐old man and a 70‐year‐old man with underlying ILD presented to our hospital with a few days of fever and respiratory symptoms after receiving the BNT162b2 vaccine. Drug‐related pneumonitis due to the COVID‐19 mRNA vaccine was diagnosed. One case was diagnosed with lymphocytic alveolitis by bronchoalveolar lavage fluid and transbronchial lung cryobiopsy. Both patients were successfully treated with corticosteroids, and they attended outpatient clinics thereafter. Although the safety and efficacy of COVID‐19 vaccines have been established, further studies are needed to estimate long‐term data and reports of rare adverse reactions. We present the clinical course of two cases, review previously published case reports on COVID‐19 mRNA vaccine‐related ILD and discuss the relevant findings.

## Graphical abstract

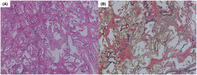

## INTRODUCTION

According to the World Health Organization, there are more than 350 million confirmed cases of coronavirus disease 2019 (COVID‐19), with more than 5.5 million deaths as of January 2022. The global morbidity, mortality and societal disruption prompted accelerated clinical vaccine development. Since December 2020, the emergency use authorization of two mRNA vaccines, BNT162b2 mRNA (Pfizer‐BioNTech) and mRNA‐1273 (Moderna), in less than a year from the emergence of severe acute respiratory syndrome coronavirus 2 (SARS‐CoV‐2) represents a landmark. A two‐dose regimen of BNT162b2 conferred 95% protection against COVID‐19 in phase III clinical trials.^1^ Furthermore, a series of reports has shown that the third dose of mRNA vaccines is effective against SARS‐CoV‐2 Omicron variant,^2^, ^3^ which will further increase the absolute number of vaccination opportunities. Local and systemic side effects are relatively common, especially after the second vaccination.^4^ These mostly include fever, malaise, headache, myalgia and arthralgia, which are only mild or moderate in severity and are limited to the first 2 days after vaccination.^4^, ^5^ In a large cohort study from Israel, BNT162b2 receipt was associated with myocarditis, lymphadenopathy, appendicitis and herpes zoster compared to the unvaccinated control group.^6^ Park et al.^7^ presented the first published case of interstitial lung disease (ILD) followed by COVID‐19 vaccination as a rare adverse disease. Herein, we report two cases of vaccine‐related ILD, one of which was confirmed histologically by cryobiopsy to have alveolitis. We present the clinical course of our cases, review previous evidence and discuss the management.

## CASE REPORT

### Case 1

From July to October 2020, a 67‐year‐old man was admitted to our department for treatment of severe COVID‐19 pneumonia requiring intubation. His body mass index was 32.5 kg/m^2^ and had hypertension and diabetes, which were well controlled with medications. He was a past smoker with no history of cardiovascular, allergic or connective tissue disease (CTD). Although there was no obvious abnormality on physical examination or serological blood tests, chest radiographs showed reticular shadows in the bilateral lower lobes. We suspected mild chronic interstitial pneumonia as an underlying disease; hence, small doses of steroids were continued, and the most recent dose was 2.5 mg of prednisolone.

**FIGURE 1:**
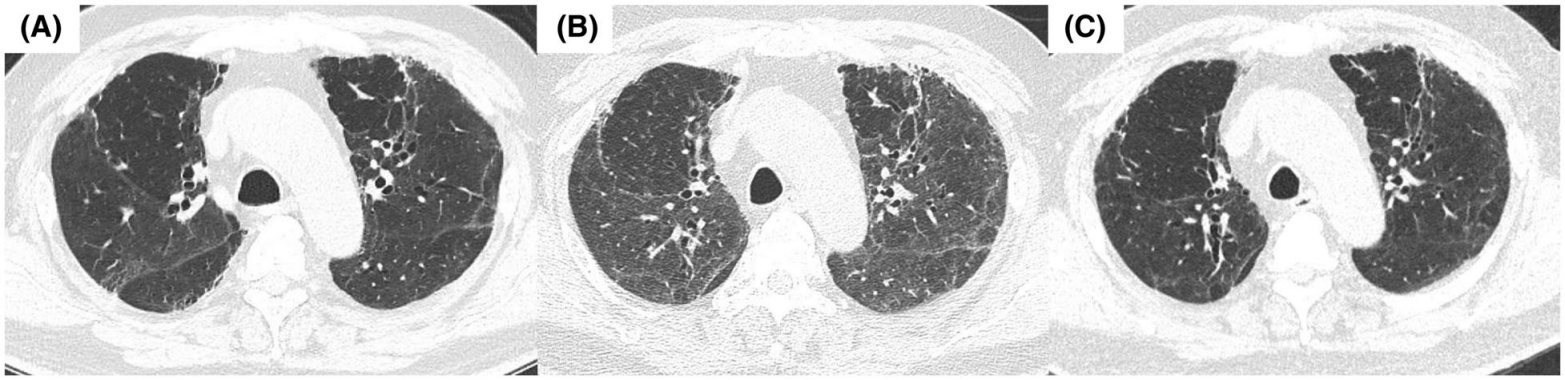
High‐resolution computed tomography (CT) images before vaccination (A: December 2020), 1 day after COVID‐19 mRNA vaccination (B: July 2021) and after treatment (C: September 2021). After vaccination, diffuse ground‐glass opacities (GGOs) were superimposed on pre‐existing reticular opacities. The GGOs on chest CT were ameliorated after the treatment

In mid‐July 2021, he presented to the pulmonary outpatient clinic with a 1‐day history of dry cough. He denied any recent changes in his living environment and exposure to chemicals or organic particles. One day before symptom onset, the first dose of the BNT162b2 mRNA vaccine had been administered. On admission, his body temperature was 37.5°C and peripheral oxygen saturation was 89% on room air. He had no rash, oedema or bilateral crackles on auscultation. Multiplex polymerase chain reaction (PCR) testing of a nasopharyngeal swab for SARS‐CoV‐2 and other common respiratory viruses was negative. An electrocardiogram showed sinus rhythm without ST changes. Blood investigations revealed elevated alveolar damage and inflammatory markers (Table 1). Brain natriuretic peptide and serum procalcitonin levels were normal. In a pulmonary function test, the forced vital capacity was 2.52 L (64.1% of predicted), reduced from 3.15 L (79.3% of predicted) 6 months before admission. Chest computed tomography (CT) revealed bilateral diffuse ground‐glass opacities (GGOs) (Figure 1B). We suspected acute interstitial pneumonia due to vaccination, acute exacerbation of chronic interstitial pneumonia and respiratory infection as the differential diagnosis.

**TABLE 1:** Clinical features of COVID‐19 mRNA vaccine‐related ILD and clinical outcomes

Case	Park et al.^7^	Yoshifuji et al.^8^	Kono et al.^9^	Shimizu et al.^10^	Shimizu et al.^10^	Shimizu et al.^10^	Matsuzaki et al.^11^	Case 1	Case 2
Age/sex	86/Male	60/Male	66/Male	66/Male	85/Male	62/Male	65/Male	67/Male	70/Male
Smoking status	Non‐smoker	Ex‐smoker	Non‐smoker	Ex‐smoker	Ex‐smoker	Non‐smoker	Ex‐smoker	Ex‐smoker	Non‐smoker
Underlying ILD	No	No	No	Yes	Yes	No	No	Yes	Yes
Onset since given vaccine	1 day after the first vaccination	2 days after the second vaccination	2 days after the second vaccination	1 day after the first vaccination	3–5 days after the first vaccination	2 days after the second vaccination	2 days after the first vaccination	1 day after the first vaccination	2 days after the second vaccination
Symptoms	Fever, dyspnoea	Dyspnoea	Fever	Fever, fatigue	Dyspnoea	Fever	Fever	Fever, dry cough	Fever, dyspnoea
RT‐PCR test for SARS‐CoV‐2 nucleic acid	Negative	Negative	Negative	Negative	Negative	Negative	Negative	Negative	Negative
Autoantibodies for CVD	Negative	Negative	Negative	Negative	Negative	MPO‐ANCA (+)	Negative	Negative	Negative
Serological tests at diagnosis									
KL‐6, U/ml	—	800.0	401.0	1306	4084	297.0	214.0	2176	274.0
SP‐D, ng/ml	—	155.0	145.0	376.4	675.5	189.0	73.1	253.6	173.0
BAL findings									
Macrophages, %	—	46.9	—	55	30.7	—	1.0	33.0	—
Lymphocytes, %	—	31.3	—	42.3	62.7	—	14.0	29.0	—
Neutrophils, %	—	21.9	—	1.7	0	—	78.0	3.0	—
Eosinophils, %	—	0	—	1	6.7	—	7.0	35.0	—
CD4/CD8	—	1.26	—	1.3	6.6	—	0.62	1.5	—
Treatment provided	mPSL 1 mg/kg	mPSL 1000 mg for 3 days followed by 1 mg/kg/day	mPSL 1000 mg for 3 days followed by PSL 0.5 mg/kg/day	None	mPSL 1000 mg for 3 days followed by PSL 1 mg/kg/day	PSL 20 mg/day	mPSL 1000 mg for 3 days followed by PSL 1 mg/kg/day	mPSL 1000 mg for 3 days followed by PSL 1 mg/kg/day	PSL 0.5 mg/kg/day
Intubation period	None	7 days	2 days	None	None	None	None	None	None
Clinical outcomes	Improved	Improved	Improved	Improved	Improved	Improved	Improved	Improved	Improved

*Note*: We defined ‘improved’ as a status in which the patient's symptoms and image findings were relieved, and the patient could be discharged.

Abbreviations: BAL, bronchoalveolar lavage; COVID‐19, coronavirus disease 2019; CVD, collagen vascular disease; ILD, interstitial lung disease; KL‐6, Krebs von den Lungen 6; MPO‐ANCA, myeloperoxidase‐anti‐neutrophil cytoplasmic antibody; mPSL, methylprednisolone; PSL, prednisolone; RT‐PCR, real‐time fluorescence polymerase chain reaction; SARS‐CoV‐2, severe acute respiratory syndrome coronavirus 2; SP‐D, surfactant protein D.

**FIGURE 2:**
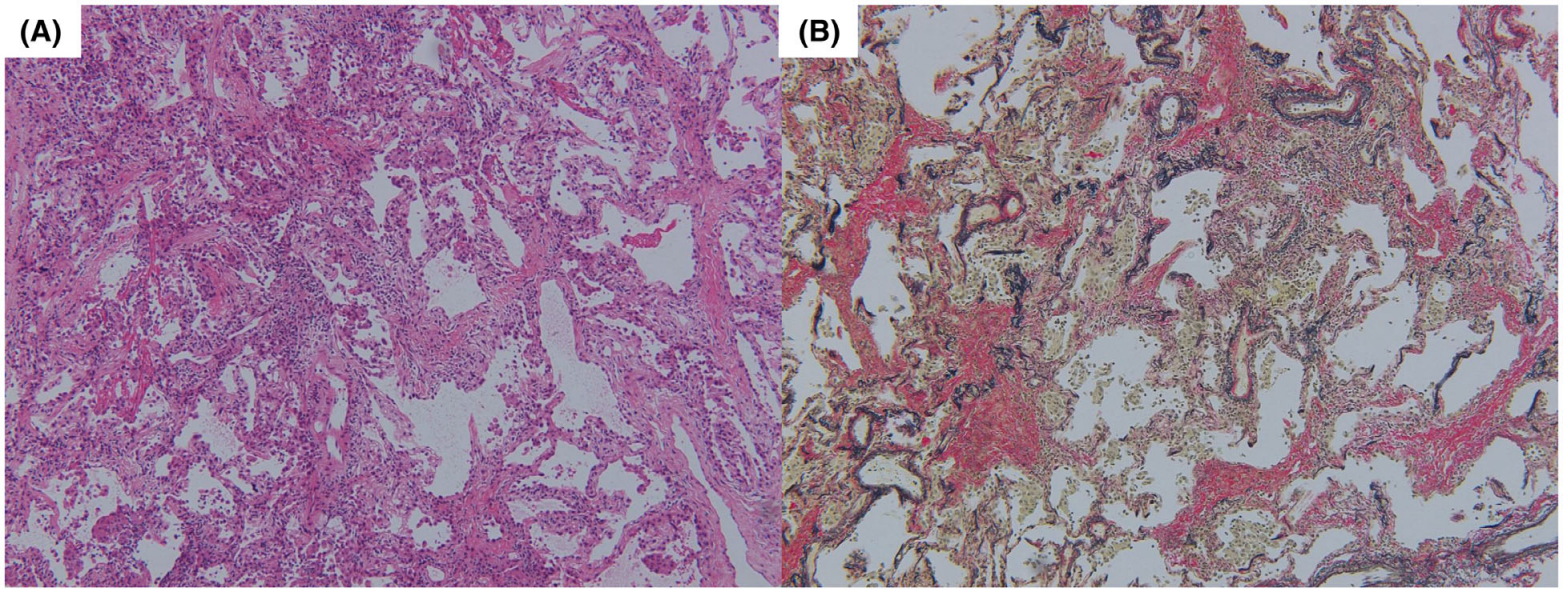
Histology of a lung specimen obtained by transbronchial lung cryobiopsy. (A) Thickening of the alveolar walls with lymphocytic infiltration (haematoxylin–eosin stain, ×200). (B) The increased collagen fibres were mottled and irregularly distributed. Against the background of these scarring changes, infiltration of inflammatory cells into alveolar walls was observed (Elastica van Gieson staining, ×100)

On day 2, we performed bronchoalveolar lavage (BAL) from the right middle lobe and transbronchial lung cryobiopsy (TBLC) from the right lower lobe. BAL fluid analysis revealed a total cell count of 8.7 × 10^5^/ml (macrophages, 33.0%; lymphocytes, 29.0%; and eosinophils, 35%). Gram stain; acid‐fast Bacillus stain; PCR test for tuberculosis, *Pneumocystis jirovecii* and *Aspergillus*; multiplex PCR for common respiratory viruses such as coronavirus, respiratory syncytial virus and cytomegalovirus; fungal stain; and bacterial culture of the BAL fluid were all negative. Histopathological examination showed alveolitis with lymphocyte infiltration (Figure 2A). The increased collagen fibres were mottled and irregularly distributed, suggesting scarring from previous lung injury caused by COVID‐19. Against the background of these scarring changes, alveolar lesions with infiltration of inflammatory cells were observed (Figure 2B). Based on the clinical course, laboratory results, radiological features and histopathological findings, we diagnosed the patient with acute lymphocytic alveolitis related with COVID‐19 mRNA vaccination. After bronchoscopy, he was treated with corticosteroids and his symptoms rapidly improved; he was discharged on day 22. CT performed 2 months after ILD onset showed an improvement of the bilateral GGO (Figure 1C).

### Case 2

**FIGURE 3:**
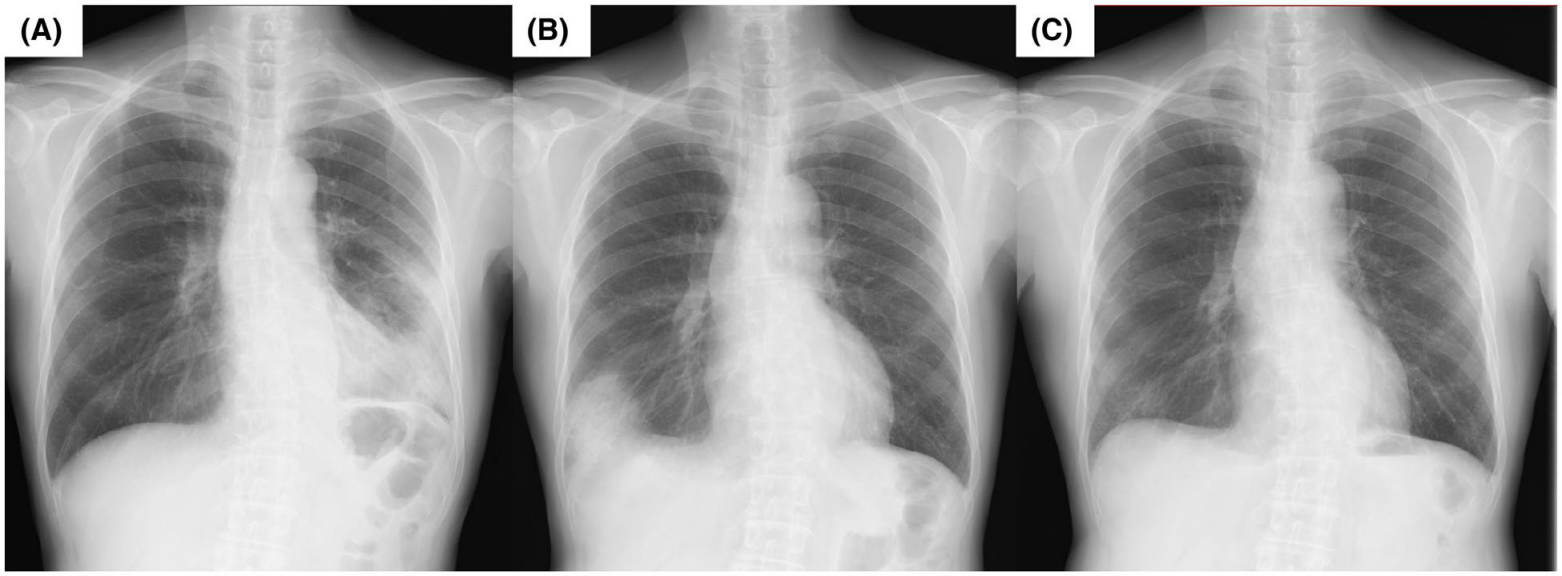
Chest radiographs before vaccination (A: September 2020), 2 days after COVID‐19 mRNA vaccination (B: July 2021) and after treatment (C: September 2021). After vaccination, infiltrative shadows in the right lower lobe were visible. These findings ameliorated after the treatment

In early September 2020, a 70‐year‐old man was admitted to our department to be examined for infiltrative shadows in the left upper and lower lobes; a SARS‐CoV‐2 PCR test was negative (Figure 3A). He had no comorbidities or allergies, and he had never smoked. BAL from the left middle lobe and TBLC from the left lower lobe were performed. He was diagnosed with cryptogenic organizing pneumonia (COP) after a multidisciplinary team discussion. After diagnosis, 0.5 mg/kg of prednisolone was started and gradually tapered until January 2021.

The first and second doses of the BNT162b2 mRNA vaccine were administered in June and July 2021, respectively. Two days after vaccination, the patient developed fever (38°C) and dyspnoea and visited the outpatient clinic. He had no cough or sputum. There were no obvious abnormalities on physical examination, and there was no evidence of infectious disease or CTD. Laboratory tests revealed normal parameters, except for lactose dehydrogenase (248 IU/L), C‐reactive protein (3.21 mg/dl) and surfactant protein D (173 ng/ml). Chest radiography revealed an infiltrative shadow in the right lower lobe (Figure 3B). Multiplex PCR testing of a nasopharyngeal swab for influenza and other common respiratory viruses was negative. We made a clinical diagnosis of vaccine‐induced COP‐like reaction. After starting 0.5 mg/kg of prednisone, his condition rapidly improved. As the symptom onset was acute after vaccination and the clinical course was transient, we believe the diagnosis is valid. Corticosteroid therapy was tapered off within 2 months of the diagnosis. Follow‐up chest radiography revealed complete improvement (Figure 3C). The patients reported in this manuscript provided written informed consent for publication.

## DISCUSSION

We report two cases of COVID‐19 mRNA vaccine‐related ILD successfully treated with corticosteroid therapy. Both had underlying ILD, and vaccine‐related ILD was pathologically confirmed in one case by cryobiopsy.

According to a few reports of influenza vaccine‐associated ILD, the clinical characteristics are as follows.^7^ The symptom onset was acute and occurred at a median of 2 days after vaccination, and fever appeared in most patients. Bilateral distribution and GGO were confirmed when chest CT findings were available. All patients recovered, and most responded well to steroid therapy. Although publication bias is a significant limitation, Asian ethnicity and ILD were thought to be risk factors for influenza vaccine‐related ILD because eight of 10 patients were Asian and two had underlying ILD.

To date, seven cases of COVID‐19 mRNA vaccine‐associated ILD have been reported in the English literature (Table 1).^7^, ^8^, ^9^, ^10^, ^11^ Symptom onset was within a few days after vaccination, and fever appeared in most cases. Two patients were intubated, but all patients responded to steroid therapy. All patients were over 60 years of age and reported from Asian countries, including Korea and Japan, and two patients had underlying ILD.

The mechanism of drug‐induced ILD is not well understood. However, two mechanisms have been proposed: cytotoxic and immune‐mediated lung injury. Cytotoxic injury to pneumocytes or the alveolar capillary endothelium may occur directly, whereas immune‐mediated reactions occur through T‐cell regulation.^12^ In our patients, immune‐mediated reaction of the lung to the vaccine was suspected, based on the lymphocytic alveolitis in the BAL fluid or TBLC and efficacy of corticosteroids, consistent with previous studies.^8^, ^10^, ^11^ Furthermore, both were Asian and had underlying ILD.

In general, management of acute exacerbations is essential in the treatment of interstitial pneumonia. In particular, acute exacerbation of idiopathic pulmonary fibrosis (IPF) is a critical condition caused by various factors, including drugs, and has attracted much attention due to its high fatality rate.^13^ A previous report highlighted the risk of acute exacerbation of IPF caused by the immune response induced by the influenza A vaccine^14^; therefore, careful monitoring is required. In the future, when proceeding with SARS‐CoV‐2 vaccination of patients with ILD, physicians should be aware of subsequent worsening of ILD, especially in IPF patients. It is difficult to determine whether the cases in this report are vaccine‐induced ILD or acute exacerbations of the underlying interstitial pneumonia. Neither of our patients had IPF and both were successfully treated with steroids.

To the best of our knowledge, this is the first reported case of pathologically confirmed ILD that occurred after COVID‐19 vaccination. As the number of SARS‐CoV‐2 vaccinations increases, physicians should always consider the possibility of vaccination‐related lung injury, if systemic or respiratory symptoms occur after vaccination. In addition, when vaccinating patients with ILD, especially those with IPF, careful follow‐up after COVID‐19 vaccination is preferable. However, the benefits of currently approved vaccines in the general population continue to strongly outweigh the risks; hence, this report does not call into question the recommendation of the vaccines. This study is a single‐centre report with a limited number of cases. Further studies are required to determine the possible adverse reactions and their risk factors.

## CONFLICT OF INTEREST

None declared.

## AUTHOR CONTRIBUTION

Conceptualization: Shinyu Izumi. Investigation: Clara So, Shinyu Izumi, Akane Ishida, Ryo Hirakawa, Yusaku Kusaba, Masao Hashimoto, Satoru Ishii, Hideki Miyazaki, Motoyasu Iikura, Masayuki Hojo. Writing—original draft: Clara So, Shinyu Izumi. Writing—review and editing: Clara So, Shinyu Izumi, Akane Ishida.

## ETHICS STATEMENT

The authors declare that appropriate written informed consent was obtained for the publication of this manuscript and accompanying images.

## Data Availability

The data that support the findings of this study are available from the corresponding author upon reasonable request.
